# Protecting Privacy of Shared Epidemiologic Data without Compromising Analysis Potential

**DOI:** 10.1155/2012/421989

**Published:** 2012-02-02

**Authors:** John Cologne, Eric J. Grant, Eiji Nakashima, Yun Chen, Sachiyo Funamoto, Hiroaki Katayama

**Affiliations:** ^1^Department of Statistics, Radiation Effects Research Foundation, 5-2 Hijiyama Park, Minami-ku, Hiroshima 732-0815, Japan; ^2^Department of Epidemiology, Radiation Effects Research Foundation, Hiroshima 732-0815, Japan; ^3^Department of Medical Informatics, Graduate School of Medicine, Dentistry and Pharmaceutical Sciences, Okayama University, 2-5-1 Shikata-cho, Okayama 700-8558, Japan; ^4^Department of Information Technology, Radiation Effects Research Foundation, Hiroshima 732-0815, Japan

## Abstract

*Objective*. Ensuring privacy of research subjects when epidemiologic data are shared with outside collaborators involves masking (modifying) the data, but overmasking can compromise utility (analysis potential). Methods of statistical disclosure control for protecting privacy may be impractical for individual researchers involved in small-scale collaborations. *Methods*. We investigated a simple approach based on measures of disclosure risk and analytical utility that are straightforward for epidemiologic researchers to derive. The method is illustrated using data from the Japanese Atomic-bomb Survivor population. *Results*. Masking by modest rounding did not adequately enhance security but rounding to remove several digits of relative accuracy effectively reduced the risk of identification without substantially reducing utility. Grouping or adding random noise led to noticeable bias. *Conclusions*. When sharing epidemiologic data, it is recommended that masking be performed using rounding. Specific treatment should be determined separately in individual situations after consideration of the disclosure risks and analysis needs.

## 1. Introduction

Scientific collaboration often necessitates sharing data with researchers outside the organization that collects and maintains the data. Sharing microdata—records of individual information on a relatively small number of subjects—carries a risk of disclosure (determining the identity of one or more individuals) and, if such disclosure occurs, subjects may be harmed through access to sensitive information contained in the data [1–4]. Subjects who supply data are understandably concerned about the possibility of their private data being disclosed. Also, despite contracts and technical implementations to ensure that data remain confidential, researchers themselves may feel uneasy about releasing private data to outside laboratories. Therefore, it makes sense to take additional steps to ease such concerns. In the United States, one approach is the Federal Certificate of Confidentiality [3]; however, national laws may be of little use when data are shared in international collaborations.

Confidentiality of research data remains an issue despite routine application of methods for protecting privacy; indeed, it has recently been shown that an individual can be identified as contributing or not to publicly available summary data if one has access to certain components of the individual's data [5]. Institutions have legal and ethical responsibilities to ensure that research subjects' privacy is protected [6, 7]. Direct identifiers (names, addresses, birth dates, etc. that have virtually one-to-one correspondence with individuals) are routinely removed and the microdata are anonymized by deleting numbers used to link to identifying information in the source database. This alone, however, does not ensure protection of privacy; the data values themselves may possess some degree of subject specificity or uniqueness that could in principle be used to identify individual subjects [8–10]. Risk of identification via the data values can be reduced by masking [11], one of several techniques useful for “statistical disclosure limitation” or “statistical disclosure control” [11–13], the goal of which is to render data safe against attackers/intruders/snoopers while retaining as much information as possible to allow effective statistical analysis [1]. A great deal of work has been done in this area, resulting in methods that allow data managers to consider the balance between disclosure risk (security) and analytical integrity (utility) [14], but the ideal of a data set safe against intrusion yet retaining its full richness for statistical analysis is elusive [13]. Nevertheless, there are few documented instances of successful attacks breaching the confidentiality of research subjects [1].

Much of the data disclosure literature speaks specifically of information organizations (or “data stewardship organizations” [1]), data managers, and clients. In such settings there will typically be substantial resources devoted to data disclosure limitation. However, we are concerned with data to be shared among public health and epidemiologic research collaborators as opposed to data considered a commodity to be made public. In the collaborative setting—where collaborators have a right to expect to be able to obtain essentially the same analytical results that would be obtained using the original data within the institution that maintains those data—the trade-off between security and utility renders it inevitable that the disseminating institution receives reasonable assurances in writing as to use of the data rather than relying solely on data masking [15]; this indeed has been demonstrated to be useful [1]. Even without such assurances, identification of research subjects via data values is unlikely [16], but there may be a perceived risk of identification by research subjects, who are concerned about psychosocial and financial risks resulting from disclosure of personal health or genetic information [17]. Some degree of masking and assessment of identifiability risk is therefore necessary: not so much as a defense against attack, but as a means of providing reasonable assurances that satisfy stakeholders [1].

There seems to be little discussion of data disclosure control in the epidemiologic literature and a dearth of practical methods for dealing with it that are accessible to epidemiologic researchers. Existing methods of disclosure control may be difficult for epidemiologic investigators to use and, unlike public release of data, the effort may not be justified by the limited amount of data or restricted sphere of dissemination (not to mention the inherently smaller risk) involved in collaborative data sharing. We therefore investigated simple steps to address disclosure concerns, with a view towards the impact on analytic utility of the masked data. We also illustrate a graphical approach to characterizing the trade-off between disclosure risk and analysis potential, which is analogous to the risk-utility (R-U) plot (also referred to as a “map”) known in the data disclosure literature [12, 18], except that it may be generated using quantities easily derived from standard methods of risk analysis and is transformed to mimic measures familiar to epidemiologists. The approach is illustrated using a small set of microdata constructed from the Japanese Atomic-bomb Survivor population [19].

## 2. Materials and Methods

Developing a data-masking strategy involves sequentially answering three questions: (1) is there a risk of identifying individuals, and if so, how great is it? (2) If the risk of identification is unacceptable, can it be reduced by masking the data? (3) If data are masked, what impact does the masking have on the results of analyses? If the impact of masking on analysis is unacceptable, it may be necessary to reevaluate the acceptable degree of identification risk, resulting in the need to seek a compromise between disclosure risk and informativeness (utility) of the data.

### 2.1. Anonymity

We consider first the risk of identifying individuals and whether masking is required. Although deletion of identifying keys is referred to as anonymization, we use the term anonymity loosely to imply that confidentiality is maintained. Also, the term “keys” can mean either the elements used to link records in a relational database or the variables that are shared in common between any two datasets.

#### 2.1.1. Identification

Identification could be absolute, based on linkage to identifying keys (names, etc.) in the institutional database, or predictive (presumed), based only on data values in the distributed dataset (using keys available to the potential intruder, such as gender, and region). With appropriate access control the institutional database should be secure from outside intrusion, and in-house researchers should not generally need to access identifying information for purposes of analysis. Source data may be separated into *resource* data, to which linkage and access are highly restricted, and *research* data, which are anonymized by removing identifying information prior to use in-house. Absolute identification is therefore not considered a plausible scenario.

In the case of distributed data, linkage to outside sources of information might in principle facilitate identification of individuals in a predictive sense (with some degree of uncertainty) based on the values in the dataset. Coarsely stratified pieces of information that are not in themselves linkable can, when combined, increase the possibility of identification [20]. Although the ability of researchers to perform such linkage may be overestimated [16], research subjects who perceive possible harm to themselves through the threat of lost anonymity may be unwilling to participate [1, 4].

#### 2.1.2. Identifiability Risk

Risk of identification in an anonymized dataset may be defined as the probability that an individual's identity is correctly inferred conditional on the masked data and other prior information available to the intruder. Because it is difficult to speculate what information might be used by an intruder to learn the identities of subjects (the “disclosure attack scenario” [13]), we used as a proxy to this scenario matching records in the distribution dataset to records in the source data, which depends on uniqueness of an individual's data [1]; a perfect link would be the epitome of disclosure, as it would reveal absolutely the identity of an individual in the microdata. We compared data values in the distribution dataset to the source database to determine whether an individual was theoretically identifiable via his or her data (“per-record measure of risk” [21]). This is purely an academic exercise that represents a worst-case scenario unlikely to be realized with typical access-control safeguards. The results will therefore overestimate actual risk [1].

An aggregate measure of risk for the entire dataset can be derived by considering uniqueness of records [22]. If only one record in the source database matches that of an individual in the distribution dataset, that individual is theoretically identifiable with probability one. Let *n*_1_ be the number of such persons in the distribution data. If two records in the source database exactly match that of an individual in the distribution data, then the probability of correctly identifying that individual is 0.5 (1 out of 2); let *n*_2_ be the number of such individuals. Continuing in this way, define an identifiability risk score as

(1) $$\begin{array}{cc} & \text{identifiability risk score}\\ & =\frac{n_{1}\times 1+n_{2}\times \left(1/2\right)+n_{3}\times \left(1/3\right)+n_{4}\times \left(1/4\right)+\cdots }{\text{total number of individuals in the distributed dataset}\;\left(N\right)}\\ & =\frac{\sum_{i=1}^{k}n_{i}/i}{N}. \end{array}$$

This is similar to a measure of identification risk described elsewhere [21], except that it is an aggregate measure for the entire microdataset obtained as a weighted average of individual uniqueness measures (the uniqueness measure being the probability 1/*i* and the weight being the number of individuals *n*_*i*_ having uniqueness 1/*i*, *i* = 1,…, *k*). The value *k* is simply the largest value of *i* that occurs in a given application. The goal is to reduce the overall identifiability risk by reducing, through data masking, the numbers of distributed data records that have nearly unique matches in the source data, that is, reduce the magnitudes of the *n*_*i*_ at small values of *i*, which have a high probability (1/*i*) of identification. We define an anonymity score as

(2) $$\begin{array}{c} \text{anonymity}\;\text{score}=1-\text{identifiability}\;\text{risk}\;\text{score}, \end{array}$$

where 0 ≤ anonymity score ≤ 1, with 0 representing unambiguous identification of all individuals and 1 representing the case that no individuals are identified.

 The aforementioned definition of overall risk is slightly different from a method that counts number of individuals in the microdata who have the same values of certain variables (thereby reducing their uniqueness) [13]. However, that perspective stems from the scenario where a potential intruder has a particular individual in mind and desires to know if that individual is present in the microdata; our perspective is that of identifying any individual in the microdata, equivalent to what has been described as someone attempting to show that the system can be breached [13]. Our aggregate score is a weighted measure, which reflects varying degrees of risk, unlike the so-called “rule of three” that treats occurrences of *i* = 1 or 2 (*i* < 3) as unsafe but weights them equally [13].

### 2.2. Masking

Numerous approaches to data masking are available [23]. We consider two broad classes that do not involve deletion or subsampling of subjects: (1) grouping and (2) randomization (adding random noise to data values). Although both classes are “perturbative” [13], we distinguish them because they pose different statistical challenges in terms of the random error induced. Grouping (or coarsening), which includes stratification, rounding, and truncation, is deterministic and results in errors of the so-called “Berkson” type that do not generally induce bias. Randomization [11] is stochastic and induces classical covariate-error bias [24]. Furthermore, even though random noise may have mean zero, it does not preserve variances or correlation coefficients [1].

#### 2.2.1. Stratification

Epidemiologic data are often stratified on age, calendar year, and geographic region. Time variables are typically grouped into five-year intervals. Exposure may also be expressed using intervals. Stratified microdata would contain individual records having stratum indicators rather than actual values of covariates. Individual outcome indicators could be grouped into broad disease or cause-of-death categories.

#### 2.2.2. Rounding

Rounding to relative precision involves using a fixed number of significant digits. Rounding to absolute precision involves rounding all data to the same decimal place and results in greater loss of information with values closer to zero. If exposure values range over several orders of magnitude or display a skewed distribution, relative precision rounding might be a more natural way of masking because it preserves approximately the same amount of statistical precision regardless of the level of exposure. Birth date and age could be rounded to the nearest tenth of a year (approximately the same as year plus month) or to the nearest integer year.

#### 2.2.3. Truncation

Truncation results in values biased towards zero. Age and birth year are frequently truncated by dropping decimal fractions (ignoring month and day). Dates can be truncated by removing the day value (sometimes day is replaced by 15). Truncating exposure data could pose serious repercussions for dose-response analyses because it introduces systematic bias.

### 2.3. Analysis Potential

Analysis potential refers to how faithfully masked data can replicate results that would be obtained using the source data. A collaborator would have little interest in data that do not produce essentially the same results as an analysis of the original data. An estimated parameter (*β*) can be evaluated in terms of its precision and bias. Bias can result from, for example, covariate error; precision plays a role in the statistical significance and variability (width of confidence intervals) of risk estimates. We examine two types of masking: (1) grouping, rounding, or randomizing a continuous exposure variable and (2) deleting or combining multiple categorical variables. For a continuous exposure, we assess analysis potential (utility) via the bias and/or loss of precision in the risk estimate from a simple dose-response analysis with various degrees of masking. A statistical measure that incorporates both bias and variance is the mean squared error (MSE = variance + bias^2^); the reciprocal of MSE has been used as a measure of utility by others [18]. Further aspects of analysis, such as the dose-response shape or effect modification, are not easily incorporated into a general approach to quantifying analysis potential and so are not considered here. Another measure of utility is the degree of overlap between confidence intervals obtained with original and masked data [12]. We have not pursued use of that measure because we find it difficult to interpret quantitatively.

 We define an analysis-potential score as

(3) $$\begin{array}{cc} & \text{analysis-potential}\;\text{score}\\ & \;=1-\frac{\text{MSE}\left(\beta_{m}\right)-\text{MSE}\left(\beta \right)}{\text{MSE}\left(\beta_{m}\right)}=\frac{\text{MSE}\left(\beta \right)}{\text{MSE}\left(\beta_{m}\right)}, \end{array}$$

where *β*_*m*_ is the risk estimate obtained using masked data and *β* is the correct risk estimate obtained using nonmasked data. This score generally takes values between 0 and 1, with 1 representing no adverse impact on analysis potential. It could conceivably take on values greater than 1 because estimates obtained using biased regression methods (such as ridge regression) can have reduced variance.

**Figure 1:**
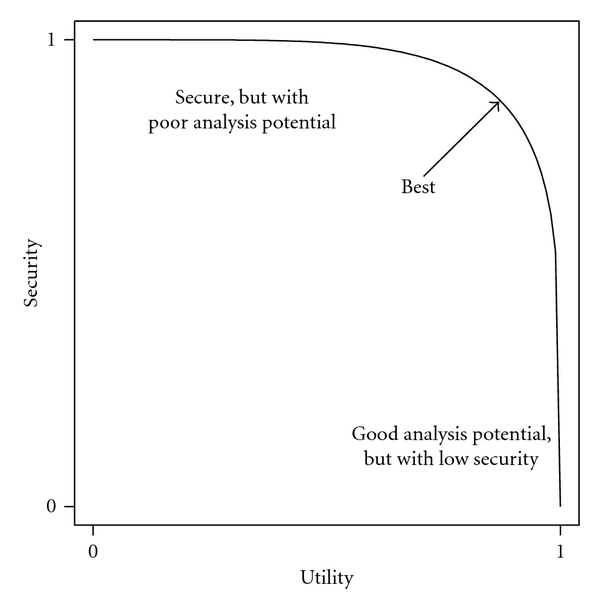
Hypothetical security-utility plot. The plot shows the trade-off between degree of masking and analysis potential. The axes are defined to allow comparison with the ideal situation of no loss of security and no loss of utility (the upper right corner).

We summarize the trade-off between anonymity and analysis potential by plotting security (anonymity score; (2)) versus utility (analysis-potential score; (3)), as shown in Figure 1. This plot is similar to a risk-utility plot (e.g., Karr et al. [12]), except that we plot security on the ordinate rather than risk, and the scales range from 0 to 1, providing some indication of where the security and utility lie relative to the ideal case of no risk and no loss of utility (analogous to an ROC curve [25]). Multiple such plots may arise from considering multiple disclosure attack scenarios.

### 2.4. Illustration

We used data from the Adult Health Study (AHS), a subset of about 23,000 persons who have participated in clinical examinations and are selected from the larger follow-up study of 120,321 Japanese Atomic-bomb Survivors and controls (the Life Span Study cohort) [26–28]. We created microdata on 64 cases of stomach cancer with radiation dose to the stomach (Gray), city, gender, age at exposure (known precisely from birth date and date of bombing), and age at diagnosis (known precisely from birth date and date of diagnosis). Radiation doses ranged from 0 to 2.74 Gray, with mean 0.61 (SD 0.74), median 0.31, and interquartile range 0.0–0.88. These doses were compared to three subsets of the larger cohort exposure database: (1) all radiation doses without regard to organ (there are multiple dose entries in a relational table, each corresponding to a different organ of the body; if the organ specifier is ignored, there are a total of 1, 830, 479 dose values), (2) stomach doses only (96, 341 values), and (3) stomach doses among the subset of persons who are members of the AHS (16, 153 values).

We estimated radiation risk using logistic regression with adjustment for uncertainties (random error) in radiation dose estimates [29]. Analyses were performed using the Epicure statistical package (HiroSoft International Corporation, Seattle, WA).

## 3. Results

### 3.1. Identifiability via Continuous Exposure

Identifiability risk scores (1) using unmasked stomach doses are shown in Table 1(a). With more information about the source of the data, larger proportions of individuals were linkable via unique dose values. These were mostly high-dose persons due to a skewed distribution of doses.

With relative precision rounding to three significant digits identifiability risk was zero. None of the cases was correctly matched (Table 1(b)), but some of the rounded dose values matched values belonging to other individuals in the source data. The proportion of such erroneous matches decreased with decreasing size of the comparison source database; the perceived identification risk based on these chance matching proportions is also shown in Table 1(b). With rounded data it would not be possible to determine whether a match was correct or erroneous, adding additional ambiguity to any presumed identification.

### 3.2. Identifiability via Multiple Categorical Variables

**Figure 2:**
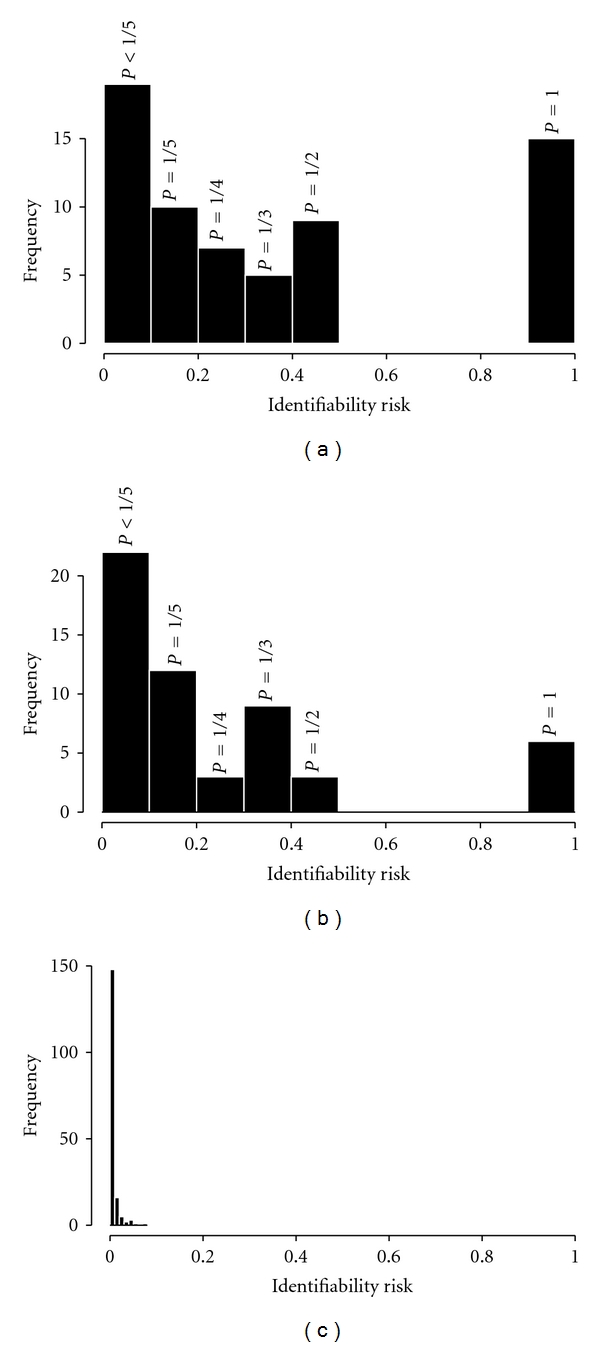
Identifiability risk with the illustration microdataset when compared to stratified source data. Data stratified on city, gender, dose, and (a) five-year intervals of age at risk and age at exposure, (b) ten-year intervals of age at risk and age at exposure, and (c) no ages included.

Figure 2(a) shows the probability of matching an individual to the clinical subset of the cohort database when data were grouped by five-year intervals of age at exposure and age at diagnosis and further broken down by dose strata, city, and gender (the strata used in analyses of the full cohort [27]). A large proportion (23%) of subjects in the microdataset were uniquely matched (*P* = 1), being the only individual occupying the corresponding stratum in the source data. The overall identification risk score (1) was 0.40. Thus, grouping the variables into categories did not afford substantial protection against matching to a unique record in the database. As the number of variables or the number of categories per variable increases, the number of persons per stratum decreases, and identifiability risk increases (recall, however, that these identifiability estimates presume access to the source data).

 Without knowledge of clinical cohort membership (i.e., comparing the distributed data to the entire cohort database), the identifiability risk was virtually zero (not shown). Thus, knowledge of a specific subcohort from which microdata were prepared may be an important factor in identification via stratified data. Using ten-year age intervals resulted in reduction by more than one-half in the numbers of cases with identification probabilities of 1.0 or 0.5 (Figure 2(b)), with an overall identification risk score of 0.23. Removing the two age variables altogether produced a large reduction in identifiability risk (Figure 2(c)). Ignoring ages, the maximum ratio of number of microdata cases to number of source-data subjects in any stratum was 0.077 and the overall identifiability risk score was 0.013. This is an overestimate of individual identifiability risk because some of the strata in the microdataset had more than one case.

### 3.3. Analysis Potential

Table 2 shows results of binary regression of the risk of stomach cancer for radiation dose, employing various forms of dose masking and using all cancer-free subjects for comparison. We include in Table 2 the results of adding random perturbations to the doses by generating random uniform deviates in the range ±0.001 Gray, ±0.01 Gray, or ±0.1 Gray. Because the results of analysis based on randomized doses are random, the results for this masking scheme are presented as the means and ranges (extreme values) from 500 repetitions.

The bias with rounding was small and there was virtually no difference between absolute and relative precision rounding, but there was a small increase in bias and standard error with stratification. With randomization, bias increased only slightly on average, but with any particular randomization the bias could be quite large, as seen by the ranges in Table 2. Randomization using ±0.1 Gray (slightly greater than 10% of the standard deviation or interquartile range) resulted in average bias of 1.6%, similar to that with stratification, but the maximum bias was as high as 6.5%. With randomization using ±0.001 Gray the bias was less than 0.1%, while using ±0.01 Gray it ranged to a little more than 0.5%.

**Figure 3:**
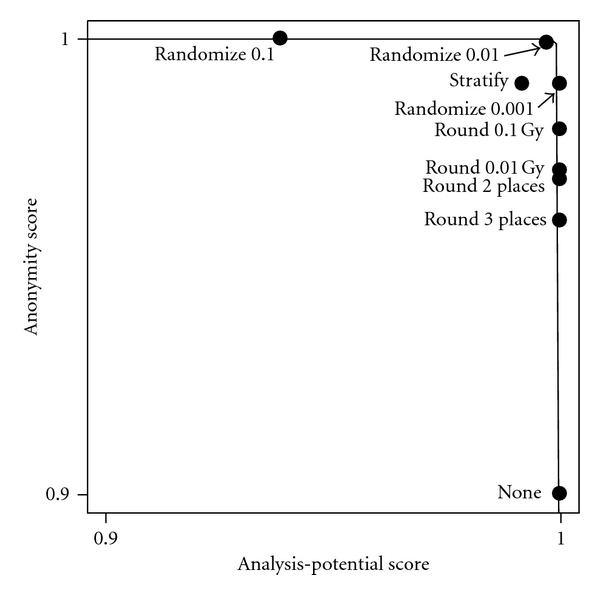
Empirical security-utility plot. The plot shows the trade-off between degree of masking and analysis potential for the example data. The points do not fall directly on the curve because the masking methods are not nested (i.e., there is not a one-to-one correspondence between degree of anonymization and analysis potential). The curve is a nonlinear regression fit of the model anonymization score = [1−(analysis-potential score)^*θ*^]^1/*θ*^ where *θ* was estimated to be 178.

Figure 3 is the plot of security versus utility for the illustration. The various masking methods led to varying degrees of reduction in identifiability risk, but most had little impact on analysis potential.

## 4. Discussion

We have adapted the risk-utility (R-U) plot (or R-U map) [12, 18] to utilize easily derived measures and to illustrate the trade-off between confidentiality and utility in a way that is familiar to epidemiologists. One could undertake a formal mathematical analysis that minimizes information loss in a masked dataset under the constraint that disclosure risk is below some allowable threshold (“minimum safety principle” [13]), although in practice it can be difficult to define information loss (predict the analytical needs of the collaborator) and quantify the risk (which depends in part on the particular attack scenario assumed). Although masked data can be analyzed appropriately given knowledge of masking mechanisms, treating the masked values as truth (ignoring the process of masking) may be the only realistic method of analysis because disclosing the masking mechanism may increase the likelihood of successful attack [23]. It would be useful to further evaluate the method using other databases with different exposure classifications and distributions to assess its generalizability. Further approaches to data masking and measures of utility are described elsewhere [12].

We found that there was little chance of identifying individuals if ages were truncated to integer values and doses in Gray were rounded to at most one decimal place of accuracy. Because the source database is highly protected and even sources of information outside the institution are subject to privacy protection laws and regulations, learning the identity of individuals in the distributed dataset seems extremely unlikely. However, in terms of analytical potential, relative-precision rounding would be preferable to absolute rounding given the wide range of magnitudes of doses.

It is prudent to consider the distributions of the variables when contemplating a masking strategy. Unique values might not be successfully masked if the magnitude of rounding is small relative to the variability in the data, particularly in sparse regions of the distribution. One could use a variable-precision rounding scheme, whereby values in sparse regions of the distribution are rounded to less precision than values in dense regions of the distribution. In the example presented here, the exposure distribution is positively skewed, so relative-precision rounding achieves the same purpose.

Masking by grouping results in Berkson-type random error, which does not introduce covariate-error bias in simple linear models but can lead to problems with more complex models, including nonlinear models and analyses of interaction with other variables [24]. Covariate-error bias in the case of classical error, such as arises with randomization of data values, leads to attenuated effect estimates (underestimation), but Berkson error can lead to overestimation of effects [30]. Thus, grouping should not be employed needlessly, and its impact on the planned analysis should be carefully considered.

The current work has several limitations in terms of generalizability. First of all, it is difficult to assess the performance of data masking without considering the specific data situation. We examined masking both continuous and categorical information and assessed identifiability for a reasonably small distribution dataset, so we think that our results should be generally applicable to most practical, small-data situations. However, the performance of any data-masking scheme should be evaluated in its own setting.

Secondly, we used an aggregate measure of identifiability and applied the same masking scheme to all individuals in the microdata. In practice, some individuals will be at higher risk of identification due to more unique values of their data (e.g., those with extreme values in a skewed distribution). Using per-record measures of disclosure risk may yield greater analysis potential if some records do not require the same degree of masking [21]. However, to achieve the same overall anonymity score may require that records with greater identifiability risk be masked to a greater extent. The resulting impact on analysis potential relative to aggregate protection deserves further investigation.

Thirdly, we did not evaluate the propensity score [31], a means of summarizing confounder information. The propensity score combines information from multiple variables into a single value, which should result in substantial masking. However, this would only be useful with variables not individually needed in analysis, such as confounders. The propensity score could not be used to replace exposures or effect modifiers of interest.

Fourthly, one caveat concerns the extent of investigation of analysis potential. Obviously, if the full analysis to be conducted by the collaborating investigator is applied to ascertain analysis potential of the masked data, there would be no need for the collaboration. Analysis potential must therefore be evaluated using simplified analyses that are expected to provide an indication of data quality without replicating the entire collaborative effort. A post hoc analysis, such as fitting the collaborators' final model to the source data, might be considered.

Finally, we have dealt with perceived (predictive) identification rather than true identification, although the latter is generally considered more relevant for purposes of disclosure control [4]. Because we assumed that identifiability is tantamount to having access to the source data, which are generally secure, we might have overestimated the true identifiability risk. However, we did not address situations where alternative, publicly available sources of data could be used in identification. As there is no way of generally characterizing all potential attack scenarios, it is best to evaluate each individual application separately, taking into account security of the source data, availability of relevant public databases, and existence of related microdatasets previously sent outside the institution.

Needs for disseminating health research data vary widely. The situation discussed here focuses on sharing microdata with a small number of investigators for research purposes, whereas medical informatics requires making data more widely available [32]. Individual data are required for purposes of developing tailor-made prognostic clinical models, and seriously perturbed data may suffer from unacceptable loss of utility. Recent calls for reproducible research would require sharing unmasked data [33]. Although we have not addressed security issues in other fields, it would be useful to establish links between collaborative and public-access scenarios. This is beyond the scope of the present paper, but would provide a useful bridge between the fields of epidemiology and medical informatics.

Issues related to privacy protection include justification (does the research contribute to “generalizable knowledge”), what constitutes “identifiable information”, and what constitutes “minimal risk” [17, 34]. Some situations may not afford sufficient masking without destroying analysis potential. For example, researchers might need unmasked dose estimates to investigate uncertainties in exposure assessment. Another situation is when subjects have a rare disease, which is not easily masked because the full population is small to begin with. Thus, in addition to masking, appropriate assurances should be obtained from the collaborators regarding privacy protection and use of the distributed data.

A recent text on molecular epidemiology notes the need for multidisciplinary collaborative research but appears to mention subject confidentiality only once in the first chapter [35]. Although actual identification of research subjects from shared data is extremely unlikely given the secure nature of research databases, it is important to consider the perceived possibility of identification. Research subjects may withhold consent if they perceive a threat to their privacy, or researchers may be concerned that such perception might impact the quality of the data provided by subjects [10]. Furthermore, laws require researchers involved in data sharing to understand and apply appropriate safeguards to protect the privacy of subjects' data [36]. Researchers should also be concerned about subjects' welfare. Article 23 of the Declaration of Helsinki of the World Medical Association states, “Every precaution must be taken … to minimize the impact of the study on their physical, mental and social integrity” [37]. This would include stigmatization caused by perceived potential for identification and its implications for the subject's health care, employment, and so forth.

## 5. Conclusions

The best masking schemes are those for which anonymity (security) and analysis potential (utility) are mutually as high as possible. Standard approaches to statistical disclosure control are developed for large-scale data releases by organizations specializing in data management but may be difficult to implement by epidemiologic researchers in small-scale data-sharing collaborations. We recommend that confidentiality be assessed by examining uniqueness of data records and attempting linkage with the source data. For disseminating microdata, we recommend that data values be rounded using relative-precision rounding. The trade-off between security and utility should be numerically evaluated in each individual data-sharing situation to assure outside researchers and study participants of the utility and security of the data. An approach to evaluating the trade-off between security and utility may be based simply on quantities easily derived by epidemiologic researchers and examined graphically as illustrated herein.

## Figures and Tables

**Table 1:** Cases in the distributed data matching to ≤3 doses in the source database.

Comparison to	Number of matches in source database (identification risk)			Overall identification risk (1)
	3 (0.33)	2 (0.5)	1 (1.0)	
	(a) Using original (nonmasked) doses			

All organ doses	2	9	18	0.39
Stomach doses only	3	5	29	0.52
AHS stomach doses	1	3	35	0.58

	(b) Using doses rounded to 3 significant digits			

All organ doses	0 (1)^a^	0 (5)	0 (6)	0 (0.14)
Stomach doses only	0	0	0 (2)	0 (0.03)
AHS stomach doses	0	0	0	0 (0)

^a^Numbers in parentheses are the numbers of values that exactly matched some entry in the source database, but in no instance was the matching individual in the source database the subject with the three-digit rounded dose.

**Table 2:** Results of fitting a linear dose response using binary regression.

Dose masking scheme	ERR^a^ (per Gray)	Standard error	Deviance	LR statistic (*P* value)	Relative bias (%)	MSE
None	0.5235	0.1548	826.36	9.27 (0.0023)	—	0.0240
Rounded to three decimal digits	0.5237	0.1548	826.35	9.28 (0.0023)	0.038	0.0240
Rounded to two decimal digits	0.5235	0.1547	826.35	9.28 (0.0023)	0	0.0239
Rounded to nearest centiGray	0.5235	0.1548	826.36	9.27 (0.0023)	0	0.0240
Rounded to nearest deciGray	0.5228	0.1547	826.36	9.27 (0.0023)	0.13	0.0239
Stratified^b^	0.5320	0.1553	826.11	9.52 (0.0020)	1.6	0.0242
Randomized^c^						
± 0.001	0.5235	0.1548	826.36	9.27	0.015	0.0240
(min, max)	(0.5234, 0.5238)	(0.1548, 0.1549)		(0.0023)	(0, 0.057)	(0.02397, 0.02398)
± 0.01	0.5239	0.155	826.36	9.28	0.16	0.0240
(min, max)	(0.5226, 0.5266)	(0.1548,0.1551)		(0.0023)	(0, 0.59)	(0.02396, 0.02407)
± 0.1	0.5271	0.155	826.33	9.31	1.6	0.0243
(min, max)	(0.5159, 0.5573)	(0.1537,0.1584)		(0.0023)	(1.4, 6.5)	(0.02415, 0.02557)

^a^ERR: excess relative risk (relative risk—1). Precision is overrepresented for comparison.

^b^Doses were stratified according to the categories used in Life Span Study Report 13 [27]. The dose value assigned to each individual was the mean of all database AHS stomach dose values in that group.

^c^A random uniform deviate between the specified range was added to the dose; if this operation resulted in a negative value, the masked dose was set to zero. Results are the averages from 500 simulations.
